# CAPMH health-related quality of life among adolescent psychiatric outpatients: a 12-month follow-up study among 12–14-year-old Finnish boys and girls

**DOI:** 10.1186/s13034-019-0278-z

**Published:** 2019-03-26

**Authors:** Anne Rissanen, Nina Lindberg, Mauri Marttunen, Harri Sintonen, Risto Roine

**Affiliations:** 1 Department of Adolescent Psychiatry, University of Helsinki and Helsinki University Hospital, Helsinki, Finland; 2 Department of Forensic Psychiatry, University of Helsinki and Helsinki University Hospital, Helsinki, Finland; 3 Department of Public Health, University of Helsinki, Helsinki, Finland; 4 Helsinki University Hospital, Administration, Research, and Development, Helsinki, Finland and Department of Health and Social Management, University of Eastern Finland, Kuopio, Finland; 5 Lohja, Finland

**Keywords:** Adolescence, Health-related quality of life, Outpatient treatment, Psychiatry

## Abstract

**Background:**

Little is known about adolescents’ perceptions about their health-related quality of life (HRQoL) in the course of routine adolescent psychiatric treatment. The aim of this 1-year follow-up study was to investigate HRQoL and changes in it among youths receiving adolescent psychiatric outpatient treatment.

**Methods:**

The study comprised 158 girls and 82 boys aged 12–14 years from 10 psychiatric outpatient clinics in one Finnish hospital district. Same-aged population controls (210 girls and 162 boys) were randomly collected from comprehensive schools. HRQoL was measured using the 16D instrument. The questionnaire was self-administered when the adolescents entered the polyclinics (= baseline), after a treatment period of 6 months, and after 12 months.

**Results:**

The mean age of respondents was 13.8 years (SD 0.63). At baseline, the mean HRQoL score of both female and male outpatients was significantly lower than that of population controls (p < 0.001). HRQoL of female patients was significantly worse than that of male patients (p < 0.001). In girls, HRQoL improved continuously during the 12-month follow-up, yet it remained worse than that of female population controls. Among boys, HRQoL was substantially better at the 6-month follow-up than at baseline, but this positive development was no longer seen at the 12-month follow-up.

**Conclusions:**

From the perspective of HRQoL, girls seem to benefit more than boys from adolescent psychiatric outpatient treatment. Possible explanations for this finding are discussed.

## Background

Adolescence is a transitional stage from childhood to adulthood during which an individual undergoes many physiological, psychological, cognitive, and social changes. Adolescence is initiated by pubertal onset and can be divided into three periods: early adolescence (12–14 years), mid-adolescence (15–16 years), and late adolescence (17–22 years) [1, 2]. Each of these periods has certain developmental tasks, including the achievement of biological and sexual maturity, the development of personal identity, the development of intimate sexual relationships, and the establishment of independence and autonomy [3].

Adolescence is a risk period for the emergence of many mental health disorders [4, 5]. This is probably related to anomalies or exaggerations of typical adolescent maturation processes acting in concert with psychosocial factors and/or biological and environmental factors [6]. The worldwide pooled prevalence of mental disorders in children and adolescents is estimated to be 13.4% [7], and approximately half of all lifetime anxiety, mood, impulse control, and substance use disorders start by the age of 14 years [8]. Externalizing disorders, such as conduct disorder and attention deficit hyperactivity disorder (ADHD), are more prevalent in boys, while internalizing disorders, such as anxiety and depressive disorders, manifest more commonly in girls [9]. Having a psychiatric disorder during childhood or adolescence is a potential risk factor for mental health problems in adulthood [8]. Although about half of young adults with a history of a psychiatric disorder in either childhood or adolescence show no psychiatric disorder in adulthood, they are at substantial risk for impairments in health, education and income, and social and family functioning as well as for crime or risk-taking behavior [10]. Thus, the years preceding adulthood are important for early detection, prevention, and treatment of psychiatric disorders.

Quality of life (QoL) is defined as “individuals’ perception of their position in life in the context of the culture and value systems in which they live and in relation to their goals, expectations, standards, and concerns” [11]. Health-related quality of life (HRQoL) can be seen as a narrower concept of QoL, as it focuses on the relationship between QoL and health status. However, in many publications these two concepts are interchangeable. HRQoL measures are increasingly used in adolescent mental health research since they provide a possibility to learn about an adolescent’s subjective perceptions and experiences of well-being. As a latent construct, HRQoL captures the ‘think’ and ‘feel’ aspects of a situation, which cannot be directly observed [12, 13]. Multidimensional HRQoL measures comprise at least physical, psychological, and social well-being dimensions in accordance with the definition of health provided by the World Health Organization (WHO) [14].

In a review by Dey et al. [15], HRQoL among children and adolescents with psychiatric disorders was compromised as compared with their healthy peers. The largest effect sizes were found for psychosocial and family-related domains and general QoL. Unfortunately, studies of this review reported mainly parents’ proxy ratings instead of the perceptions of children and adolescents themselves. Recently, Jonsson et al. [16] identified QoL studies conducted among children and adolescents who suffered from diagnosed mental or behavioral disorders. In line with the results of Dey et al. [15], the patients showed reduced self- and parent-rated QoL compared with typically developing adolescents or adolescents with other health conditions.

HRQoL serves as a general mental health and well-being outcome measure in treatment studies among adolescents [13, 17, 18]. In a study by Granö et al. [19], a need-adapted, family- and community-oriented intervention model improved HRQoL of help-seeking adolescents with mental health problems. A significant improvement in QoL was also seen in a study investigating the treatment outcome of inpatient psychotherapy among personality disordered adolescents [20] and in a study exploring adolescent mentalization-based integrative treatment among adolescents with anxiety, depression, or psychotic symptoms [21]. Recently, an intervention model derived from psychodynamic, milieu, and cognitive therapies was shown to improve QoL in adolescents with different psychiatric diagnoses [22].

Follow-up studies focusing on the HRQoL in adolescents with mental health problems are still scarce. Yet, professionals working in the field of adolescent psychiatry would benefit from this information when trying to improve the quality and content of care. The aim of this study was to investigate how early adolescents evaluate their HRQoL when entering municipal psychiatric outpatient treatment and after treatment periods of 6 and 12 months. Furthermore, we examined whether gender differences in HRQoL exist. We hypothesized that (1) adolescent psychiatric outpatients would have substantially lower HRQoL scores than their counterparts in the general population, (2) HRQoL scores would improve with psychiatric treatment, and (3) some gender differences would emerge in HRQoL scores. As a post hoc analysis, we evaluated whether being on the waiting list for treatment would improve one’s subjective HRQoL scores.

## Subjects and methods

### Setting

The data were collected in the Hospital District of Helsinki and Uusimaa (HUS), which serves approximately 1.5 million inhabitants of Southern Finland, nearly 100,000 of whom are 13–17 years old. HUS provides municipal secondary and tertiary healthcare services and comprises five hospital areas. This study was conducted in one of them, the Helsinki University Hospital area, which has altogether 11 psychiatric outpatient clinics for adolescents. Referrals to the specialized services of the hospital come from primary healthcare services, including school healthcare, social services, and health centers, as well as from private physicians. Municipal adolescent psychiatric outpatient treatment typically consists of diagnostic workups by a multiprofessional team, including a psychiatrist, a psychologist, a psychiatric nurse, an occupational therapist, and a social worker, psychoeducation, psychotherapeutic interventions, psychiatric medication, parents’ appointments, and networking with schools and child welfare services.

### Subjects

As part of a large trial focusing on the effectiveness of various secondary care interventions, we evaluated HRQoL among adolescents aged 12–14 years who were referred to 10 of the above-mentioned 11 adolescent psychiatric outpatient clinics between April 2008 and December 2009.

Same-aged pupils randomly collected from 13 comprehensive schools in Helsinki in 2013 served as population controls. Altogether 1635 pupils were invited to participate; 373 (210 girls and 162 boys, 22.8%) subsequently participated. The mean age of respondents was 14.2 years (SD 1.01).

### Measurement

HRQoL was evaluated by using the generic 16D© HRQoL instrument for adolescents aged 12–15 years [23]. The structure of the standardized 16D is based on the 15D instrument designed for adults [24]. The 16D is a self-administered questionnaire and can be used both as a profile and as a single index utility score measure [25]. It consists of 16 multiple choice questions, each representing one dimension of health (vitality, seeing, breathing, distress, hearing, sleeping, eating, discomfort and symptoms, speech, physical appearance, school and hobbies, moving, friends, mental function, excretion, depression). For each dimension, the respondent is advised to choose one of the five levels best describing his/her state of health at that moment (best level = 1, worst level = 5). The valuation system of the 16D is based on an application of the multi-attribute utility theory. A set of utility or preference weights, elicited from the public through a 2-stage valuation procedure, is used to generate the dimension level values on a 0–1 scale for each dimension (1 = no problems on the dimension, 0 = being dead) and in an additive aggregation formula the utility score, i.e. the 16D score (single index number) over all the dimensions on a 0–1 scale (1 = no problems on any dimension, 0 = being dead) [23]. Missing data were imputed by regression models according to the 15D instructions [26].

### Procedure

Adolescents and their guardians were invited to participate by mailing them information about the study project, the questionnaire, and an informed consent form as soon as their referral for adolescent psychiatric treatment had been received and accepted. Adolescents who were referred to receive crisis intervention were excluded. One reminder was sent if there was no response to the first invitation. An informed consent was obtained from both the adolescent and his/her parent or legal guardian. If the interval between answering the baseline questionnaire and the first visit to the outpatient unit exceeded 3 weeks, an additional questionnaire (baseline 2) was sent just before the visit. Both the 6- and 12-month follow-up questionnaires were mailed to adolescents who had returned the first baseline questionnaire (baseline 1). If needed, one reminder was sent to those not responding to the follow-up questionnaires.

### Background variables, diagnosis, and costs

Age and gender of the patient were recorded from the referral form. To study the intensity of treatment received, direct costs of all treatment interventions provided by HUS during a 24-month follow-up starting from referral receipt date were collected from the Ecomed^®^ clinical patient administration system (Datawell Ltd., Espoo, Finland). The same system also provided the primary clinical psychiatric diagnoses of the patients based on ICD-10 [27]. Of the up to five diagnoses that can be recorded in the system, the first one was deemed to provide the most important reason for the treatment and was thus regarded as the primary diagnosis. The psychiatric diagnoses were later aggregated into diagnostic categories according to ICD-10.

### Ethics

The study protocol was approved by the Institutional Ethics Committee of HUS on January 17, 2008 (registration number 538/E0/02). The trial was registered in the HUS Clinical Trials Register [28] with the unique trial identifier 75370.

### Statistical analyses

Data were analyzed using the SPSS for Windows statistical software version 23.0 (SPSS, Inc., Chicago, IL, USA). Comparisons between adolescents who agreed to participate and those who did not, as well as gender comparisons were performed using Student’s independent samples *t* test or the Mann–Whitney U-test, where appropriate. When comparing percentage distributions between the groups, χ^2^-test was used. Comparisons between patients and controls were performed using Student’s independent samples t-test and Mann–Whitney U-test. Comparisons between baseline and 6- and 12-month follow-up points were analyzed with repeated measures analysis of variance, followed by Bonferroni corrections. p-values < 0.05 were considered statistically significant.

## Results

The baseline 16D questionnaire was sent to 645 adolescents, 240 (158 girls and 82 boys, 37.2%) of whom filled it in and returned it. Four questionnaires were excluded because the person never visited the outpatient clinic. Of those who answered at baseline, 177 (75.0%) returned either the 6- or 12-month follow-up questionnaire, and 115 (79 girls and 36 boys, 48.7%) returned both follow-up questionnaires. Altogether 108 adolescents had to wait for their first visit for more than 3 weeks, and thus, were also sent the baseline 2 questionnaire. Of these adolescents, 72 (51 girls and 21 boys, 66.7%) filled it in.

### Attrition analysis

The age of respondents did not significantly differ from that of non-respondents (13.8 years [SD 0.63] vs. 13.7 years [SD 0.69], p = 0.129). The group of respondents comprised significantly more girls than the group of non-respondents (66.1% vs. 48.9%, p < 0.001). Respondents showed slightly higher direct treatment costs than non-respondents, but the difference did not reach statistical significance (median 6648 € [interquartile range, IRQ 2988–11706] vs. 4949 € [IRQ 1984–11929], p = 0.051). No significant differences in diagnostic categories were present between respondents and non-respondents (p = 0.169). The three most common diagnostic categories were behavioral and emotional disorders with onset usually occurring in childhood or adolescence (F90–98) (respondents: 32.2% vs. non-respondents: 33.9%), affective disorders (F30–39) (25.4% vs. 21.0%), and neurotic, stress-related, and somatoform disorders (F40–48) (17.8% vs. 18.8%). The prevalence of persons encountering health services for examination and investigation (Z00–Z13) was 9.7% among respondents and 16.4% among non-respondents.

### Comparisons of population controls and patients regarding background variables

Population control subjects were slightly older than patients (14.2 years [SD 1.01] vs. 13.9 years [SD 0.63], p < 0.001). Further, the population control group comprised significantly less girls (56.3% vs. 66.1%, p = 0.016).

### Comparisons of population controls and patients regarding HRQoL scores

**Fig. 1:**
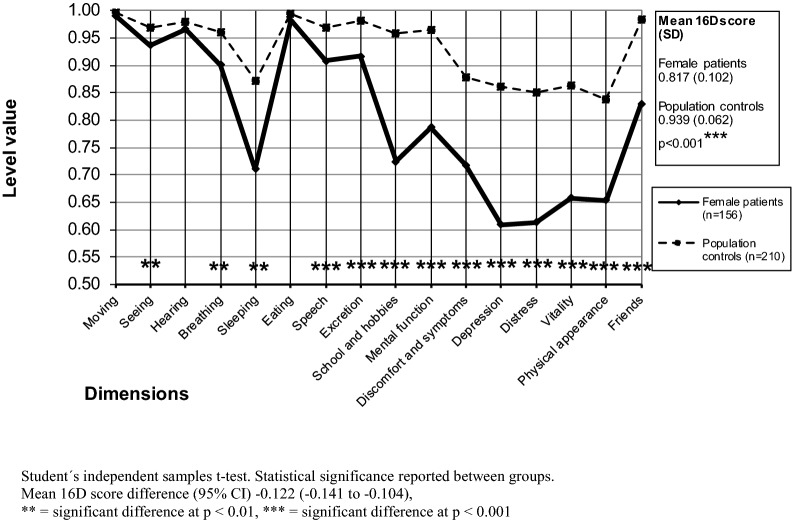
Mean baseline 16D profiles of the female outpatients and their controls

**Fig. 2:**
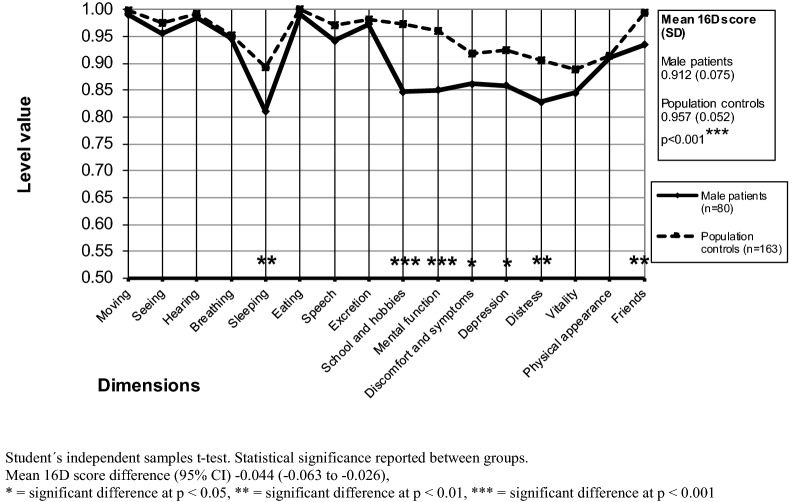
Mean baseline 16D profiles of the male outpatients and their controls

Compared with controls, both female and male patients showed a significantly lower mean 16D score (p < 0.001) (Figs. 1, 2, Table 1). Focusing on dimensions, female patients were significantly worse off than their community peers on 13 of the 16 dimensions (seeing, breathing, sleeping, speech, excretion, school and hobbies, mental function, discomfort and symptoms, depression, distress, vitality, physical appearance, friends) (Fig. 1), whereas male patients were significantly worse off than their controls on 7 dimensions (sleeping, school and hobbies, mental function, discomfort and symptoms, depression, distress, friends) (Fig. 2).

**Table 1:** The baseline 16D dimensions and score of outpatients and population controls

Variable	Baseline				Population				p-value					
	Female patients (n = 156)		Male patients (n = 80)		Population, females (n = 210)		Population, males (n = 163)		(a)vs.(d)	[a]vs.[d]	(b)vs.(f)	[b]vs.[f]	(a)vs.(b)	[a]vs.[b]
	(a)	[a]	(b)	[b]	(d)	[d]	(f)	[f]						
	Mean (SD)	Median [IQR]	Mean (SD)	Median [IQR]	Mean (SD)	Median [IQR]	Mean (SD)	Median [IQR]	S	M–W	S	M–W	S	M–W
Moving	0.991 (0.058)	1.000 [1.000–1.000]	0.991 (0.058)	1.000 [1.000–1.000]	0.997 (0.036)	1.000 [1.000–1.000]	0.998 (0.028)	1.000 [1.000–1.000]	0.263	0.230	0.313	0.212	0.977	0.976
Seeing	0.937 (0.113)	1.000 [0.769–1.000]	0.956 (0.115)	1.000 [1.000–1.000]	0.968 (0.080)	1.000 [1.000–1.000]	0.976 (0.781)	1.000 [1.000–1.000]	0.003	0.004	0.175	0.145	0.223	0.108
Hearing	0.966 (0.094)	1.000 [1.000–1.000]	0.985 (0.064)	1.000 [1.000–1.000]	0.979 (0.079)	1.000 [1.000–1.000]	0.993 (0.046)	1.000 [1.000–1.000]	0.155	0.107	0.354	0.297	0.067	0.103
Breathing	0.903 (0.200)	1.000 [1.000–1.000]	0.946 (0.142)	1.000 [1.000–1.000]	0.961 (0.110)	1.000 [1.000–1.000]	0.951 (0.126)	1.000 [1.000–1.000]	0.001	0.002	0.798	0.941	0.058	0.102
Sleeping	0.711 (0.215)	0.699 [0.471–1.000]	0.811 (0.198)	0.699 [0.699–1.000]	0.872 (0.171)	1.000 [0.699–1.000]	0.893 (0.164)	1.000 [0.699–1.000]	< 0.001	< 0.001	0.002	0.001	0.001	< 0.001
Eating	0.982 (0.086)	1.000 [1.000–1.000]	0.990 (0.060)	1.000 [1.000–1.000]	0.995 (0.045)	1.000 [1.000–1.000]	1.000 (0.000)	1.000 [1.000–1.000]	0.090	0.075	0.159	0.043	0.415	0.448
Speech	0.908 (0.173)	1.000 [1.000–1.000]	0.942 (0.134)	1.000 [1.000–1.000]	0.969 (0.098)	1.000 [1.000–1.000]	0.972 (0.098)	1.000 [1.000–1.000]	< 0.001	< 0.001	0.082	0.050	0.095	0.161
Excretion	0.917 (0.176)	1.000 [1.000–1.000]	0.973 (0.105)	1.000 [1.000–1.000]	0.981 (0.088)	1.000 [1.000–1.000]	0.982 (0.096)	1.000 [1.000–1.000]	< 0.001	< 0.001	0.525	0.381	0.003	0.010
School and hobbies	0.724 (0.246)	0.665 [0.665–1.000]	0.847 (0.221)	1.000 [0.665–1.000]	0.958 (0.127)	1.000 [1.000–1.000]	0.972 (0.101)	1.000 [1.000–1.000]	< 0.001	< 0.001	< 0.001	< 0.001	< 0.001	< 0.001
Mental function	0.787 (0.231)	1.000 [0.607–1.000]	0.850 (0.205)	1.000 [0.607–1.000]	0.965 (0.120)	1.000 [1.000–1.000]	0.961 (0.117)	1.000 [1.000–1.000]	< 0.001	< 0.001	< 0.001	< 0.001	0.034	0.050
Discomfort and symptoms	0.717 (0.213)	0.641 [0.641–1.000]	0.862 (0.205)	1.000 [0.641–1.000]	0.878 (0.185)	1.000 [0.641–1.000]	0.918 (0.157)	1.000 [1.000–1.000]	< 0.001	< 0.001	0.033	0.036	< 0.001	< 0.001
Depression	0.609 (0.255)	0.651 [0.461–0.651]	0.859 (0.209)	1.000 [0.651–1.000]	0.861 (0.208)	1.000 [0.651–1.000]	0.924 (0.157)	1.000 [1.000–1.000]	< 0.001	< 0.001	0.015	0.012	< 0.001	< 0.001
Distress	0.613 (0.230)	0.687 [0.496–0.687]	0.828 (0.191)	1.000 [0.687–1.000]	0.851 (0.193)	1.000 [0.687–1.000]	0.905 (0.163)	1.000 [0.687–1.000]	< 0.001	< 0.001	0.002	0.001	< 0.001	< 0.001
Vitality	0.681 (0.248)	0.698 [0.497–1.000]	0.844 (0.204)	1.000 [0.698–1.000]	0.864 (0.191)	1.000 [0.698–1.000]	0.889 (0.166)	1.000 [0.698–1.000]	< 0.001	< 0.001	0.091	0.118	< 0.001	< 0.001
Physical appearance	0.654 (0.278)	0.689 [0.494–1.000]	0.911 (0.178)	1.000 [1.000–1.000]	0.838 (0.217)	1.000 [0.698–1.000]	0.915 (0.160)	1.000 [1.000–1.000]	< 0.001	< 0.001	0.871	0.915	< 0.001	< 0.001
Friends	0.830 (0.227)	1.000 [0.616–1.000]	0.935 (0.151)	1.000 [1.000–1.000]	0.984 (0.080)	1.000 [1.000–1.000]	0.995 (0.042)	1.000 [1.000–1.000]	< 0.001	< 0.001	0.001	< 0.001	< 0.001	< 0.001
16D score	0.817 (0.102)	0.818 [0.752–0.898]	0.912 (0.075)	0.930 [0.868–0.983]	0.939 (0.062)	0.956 [0.912–0.985]	0.957 (0.052)	0.972 [0.937–1.000]	< 0.001	< 0.001	< 0.001	< 0.001	< 0.001	< 0.001

*SD* standard deviation, *IQR* interquartile range, *S* Student’s independent samples t-test, *M–W* Mann–Whitney U-test, statistical significance reported between groups

### Comparisons of female and male patients regarding background variables

Female patients were slightly older than male patients (14.0 years [SD 0.62] vs. 13.8 years [SD 0.62], p = 0.041) and they showed significantly higher direct treatment costs (median 7248 € [IRQ 3572–13082] vs. 4966 € [IRQ 1813–8630], p = 0.009). There were significant gender differences in diagnostic categories (p < 0.001), with girls less often showing childhood or adolescent onset behavioral and emotional disorders (F90–98) (31.1% vs. 66.1%), but more often showing affective disorders (41.2% vs. 18.6%) and neurotic, stress-related, and somatoform disorders (27.7% vs. 15.3%).

### Comparisons of female and male patients regarding baseline HRQoL scores

**Fig. 3:**
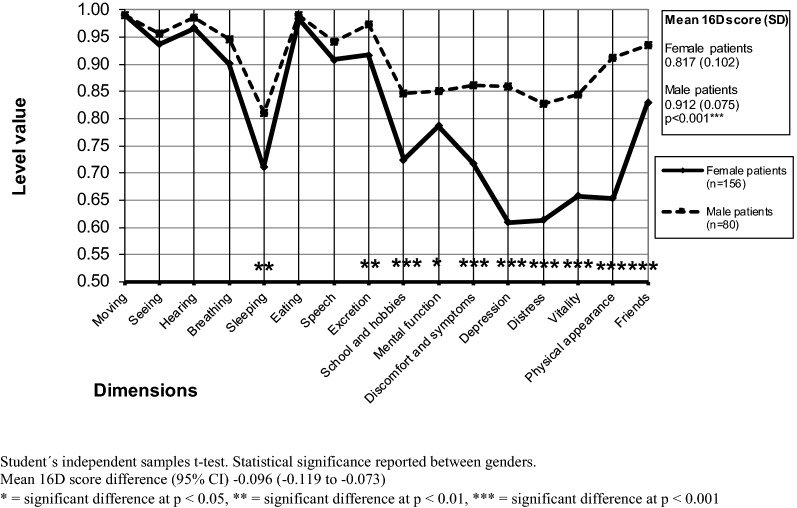
Mean baseline 16D profiles of female and male outpatients

The mean baseline 16D score of female patients was significantly lower than that of male patients (p < 0.001) (Fig. 3, Table 1). Focusing on dimensions, female patients were significantly worse off than male patients on 10 dimensions (sleeping, excretion, school and hobbies, mental function, discomfort and symptoms, depression, distress, vitality, physical appearance, friends).

#### Change in HRQoL during the follow-up period

**Fig. 4:**
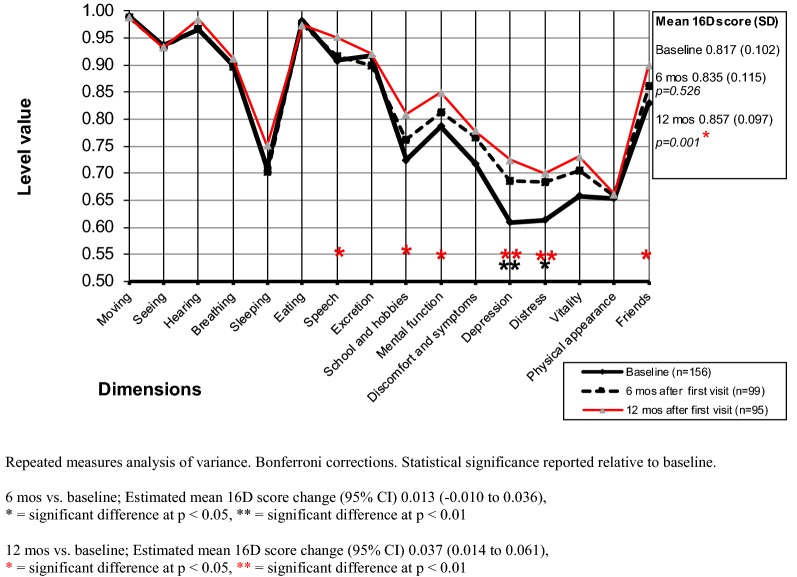
Mean baseline and follow-up 16D profiles of the female outpatients

**Fig. 5:**
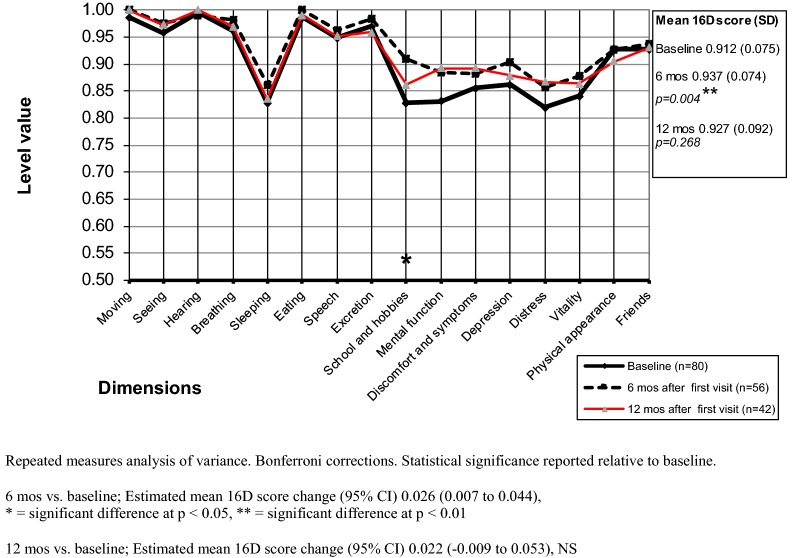
Mean baseline and follow-up 16D profiles of the male outpatients

In female patients, the mean 16D score had at the 6-month follow-up improved, but the difference was not significant (p = 0.526) (Fig. 4). However, the mean 16D score at the 12-month follow-up was significantly higher than at baseline (p = 0.001). In male patients, the mean 16D score was significantly higher (p = 0.004) at the 6-month follow-up (Fig. 5), but at the 12-month follow-up the mean 16D score of male patients no longer differed significantly (p = 0.268) from that observed at baseline.

In girls, significantly improved dimensions at the 6-month follow-up were depression and distress. In boys, significantly improved dimensions were school and hobbies. At the 12-month follow-up, significantly improved dimensions in girls were depression, distress, speech, school and hobbies, mental function, and friends, but in boys none of the dimensions differed significantly at the 12-month follow-up from that observed at baseline.

#### Adolescents on the waiting list

The mean baseline 16D score and the mean baseline 2 score did not significantly differ from each other (p = 0.124, 95% CI − 0.028 to 0.003). However, the dimension of distress improved significantly during the waiting period (p = 0.016).

## Discussion

The aim of this study was to investigate how early adolescents with mental health problems evaluate their HRQoL when entering municipal psychiatric outpatient treatment (i.e. at baseline) and 6 and 12 months after start of treatment. We also determined whether gender differences in the above exist.

As hypothesized, adolescents entering psychiatric outpatient units showed substantially impaired HRQoL relative to population controls. This was observed among both genders. The finding is in line with earlier studies in both children and adolescents [15, 16, 29–33]. Further, and again in line with earlier findings [31], adolescent patients, especially girls, reported substantial problems on psychological, social, and physical dimensions of HRQoL.

When entering psychiatric treatment, boys’ evaluation of their HRQoL was substantially better than that of girls. This agrees with some earlier QoL studies among children and adolescents [34, 35]. The finding might be explained by gender differences in psychopathology, but it might also be explained by the fact that adolescent girls are ahead of boys in their social-cognitive development [36]. It is also known that adolescent girls express better self-observation readiness than boys. For example, studies using the Youth Self-Report (YSR) instrument by Achenbach and Rescorla [37] have repeatedly found that girls report more problems in their emotional and behavioral functioning than boys [38, 39].

Our hypothesis that HRQoL would improve during follow-up was only partially supported. In girls, HRQoL improved continuously during the 12-month follow-up, yet it remained worse than that of female population controls. However, in boys, this kind of development was not observed. Their HRQoL was substantially better at 6 months than at baseline, but this positive development was no longer present at 12 months. Unfortunately, we had no information related to individual treatment plans and their realization, and, because of this, it is difficult to determine whether the poorer treatment response in boys is a consequence of a lack of effective treatment or poor treatment compliance. However, boys suffered substantially more often from externalizing disorders, whereas girls suffered from internalizing disorders. The national current care guideline on depression was introduced already in 2004 [40], and professionals in Finnish adolescent psychiatric care have been able to offer evidence-based treatment interventions to patients with depressive disorders, but a national guideline on conduct disorders was published in 2018 [41]. Thus, male patients may have received less effective treatment interventions than female patients. On the other hand, median direct treatment costs of boys were markedly lower than those of girls, indicating that either treatment of girls was substantially more intensive or boys did not adhere to treatment as well as girls. Interestingly, a recent study focusing on help-seeking behavior among Finnish adolescent boys concluded that their mental health service use is low despite their considerable needs [42]. Also, gender differences existed in expression of emotions, with adolescent girls showing more positive emotions than boys [43]. It is known that positive emotion expression contributes to both prosocial development and well-being [44, 45]. Thus, it might be that girls, with better emotion expression, have an easier time building and maintaining therapeutic relationships, which, in turn, lead to better treatment outcomes. According to findings in adolescent psychiatric acute care [46], boys seem to benefit from identification of the problem and girls from commitment to follow-up and treatment alliance. The reasons underlying our findings should be explored in future studies, and these gender differences should be taken into consideration in everyday clinical work.

Our post hoc analysis revealed that being on the waiting list decreased adolescents’ distress. Thus, expectations of psychiatric treatment appear to generate hope during the waiting period.

### Study strengths and limitations

An obvious strength of this study is that it reports adolescents’ own perceptions of their QoL. This is important since it has previously been shown that proxy HRQoL ratings by parents correlate weakly, or at best moderately, with ratings of their offspring [22, 47]. The study instrument used was originally developed for early adolescents and it has good psychometric properties [23]. The patient sample came from municipal adolescent psychiatric outpatient clinics, thus representing “ordinary patients receiving routine treatment”. We were able to use a fairly large control sample of school-going adolescents studied using the same instrument as our patients. Substantial limitations of our study were that the patient data remained relatively small and the number of drop-outs during the follow-up was high. Unfortunately, this is a well-known drawback of follow-up studies among adolescent populations [48, 49]. The fact that respondents had slightly higher healthcare costs, even though this difference did not reach statistical significance and no significant difference was seen in diagnostic categories, may indicate that they suffered from more serious psychosocial problems than the non-respondents. The school sample comprised fewer girls than the outpatient sample, and pupils were slightly older than outpatients. Furthermore, the patient data were collected approximately 4–5 years earlier than the school data, and therefore, a cohort effect, although not likely, cannot be completely ruled out. Finally, all respondents were 12–14 years old, and the findings cannot be generalized to other age groups.

## Conclusions

From the perspective of HRQoL, girls benefit more than boys from adolescent psychiatric outpatient treatment.

## Abbreviations

CI: confidence interval
HRQoL: health-related quality of life
HUS: Hospital District of Helsinki and Uusimaa
ICD-10: International Classification of Diseases, 10th edition
IRQ: interquartile range
QOL: quality of life
SD: standard deviation
WHO: World Health Organization

## References

[1] Blos P (1962). On adolescence. A psychoanalytic interpretation.

[2] Richter SK, Noshpitz JD, Flaherty LT, Sarles RM (1997). Overview of normal adolescent development. Handbook of child and adolescent psychiatry.

[3] Christie D, Viner R (2005). Adolescent development. BMJ.

[4] Kim-Cohen J, Caspi A, Moffitt TE, Harrington H, Milne BJ, Poulton R (2003). Prior juvenile diagnoses in adults with mental disorder: developmental follow-back of a prospective-longitudinal cohort. Arch Gen Psychiatry.

[5] Kessler RC, Demler O, Frank RG, Olfson M, Pincus HA, Walters EE, Wang P, Wells KB, Zaslavsky AM (2005). Prevalence and treatment of mental disorders, 1990 to 2003. N Engl J Med.

[6] Paus T, Keshavan M, Giedd JN (2008). Why do many psychiatric disorders emerge during adolescence?. Nat Rev Neurosci.

[7] Polanczyk GV, Salum GA, Sugaya LS, Caye A, Rohde LA (2015). Annual research review: a meta-analysis of the worldwide prevalence of mental disorders in children and adolescents. J Child Psychol Psychiatry..

[8] Copeland WE, Adair CE, Smetanin P, Stiff D, Briante C, Colman I, Fergusson D, Horwood J, Poulton R, Costello EJ, Angold A (2013). Diagnostic transitions from childhood to adolescence to early adulthood. J Child Psychol Psychiatry..

[9] Duinhof EL, Stevens G, van Dorsselaer S, Monshouwer K, Vollebergh WAM (2015). Ten-year trends in adolescents’ self-reported emotional and behavioral problems in the Netherlands. Eur Child Adolesc Psychiatry..

[10] Costello EJ, Maughan B (2015). Annual research review: optimal outcomes of child and adolescent mental illness. J Child Psychol Psychiatry..

[11] World Health Organization (1997). Division of Mental Health and Prevention of Substance Abuse. WHOQOL: Measuring Quality of Life MNH/PSF/97.4.

[12] Coghill D, Danckaerts M, Sonuga-Barke E, Sergeant J, ADHD European Guidelines Group (2009). Practitioner review: quality of life in child mental health–conceptual challenges and practical choices. J Child Psychol Psychiatry..

[13] Ravens-Sieberer U, Karow A, Barthel D, Klasen F (2014). How to assess quality of life in child and adolescent psychiatry. Dialogues Clin Neurosci..

[14] Bullinger M (2002). Assessing health related quality of life in medicine. An overview over concepts, methods and applications in international research. Restor Neurol Neurosci..

[15] Dey M, Landolt MA, Mohler-Kuo M (2012). Health-related quality of life among children with mental disorders: a systematic review. Qual Life Res..

[16] Jonsson U, Alaie I, Lofgren Wilteus A, Zander E, Marschik PB, Coghill D, Bolte S (2017). Annual research review: quality of life and childhood mental and behavioural disorders—a critical review of the research. J Child Psychol Psychiatry..

[17] Deighton J, Croudace T, Fonagy P, Brown J, Patalay P, Wolpert M (2014). Measuring mental health and wellbeing outcomes for children and adolescents to inform practice and policy: a review of child self-report measures. Child Adolesc Psychiatry Ment Health..

[18] Kwan B, Rickwood DJ (2015). A systematic review of mental health outcome measures for young people aged 12 to 25 years. BMC Psychiatry..

[19] Granö N, Karjalainen M, Edlund V, Saari E, Itkonen A, Anto J, Roine M (2013). Changes in health-related quality of life and functioning ability in help-seeking adolescents and adolescents at heightened risk of developing psychosis during family- and community-oriented intervention model. Int J Psychiatry Clin Pract..

[20] Feenstra DJ, Laurenssen EMP, Hutsebaut J, Verheul R, Busschbach JJV (2014). Predictors of treatment outcome of inpatient psychotherapy for adolescents with personality pathology. Person Ment Health..

[21] Griffiths H, Noble A, Duffy F, Schwannauer M (2017). Innovations in practice. Evaluating clinical outcome and service utilization in an AMBIT-trained Tier 4 child and adolescent mental health service. Child Adolesc Ment Health..

[22] Katzenschlager P, Fliedl R, Popow C, Kundi M (2018). Quality of life and satisfaction with inpatient treatment in adolescents with psychiatric disorders: a comparison between patients’, parents’, and caregivers’ (self-)assessments at admission and discharge. Neuropsychiatr..

[23] Apajasalo M, Sintonen H, Holmberg C, Sinkkonen J, Aalberg V, Pihko H, Siimes MA, Kaitila I, Makela A, Rantakari K, Anttila R, Rautonen J (1996). Quality of life in early adolescence: a sixteen-dimensional health-related measure (16D). Qual Life Res.

[24] The 15D instrument. http://www.15d-instrument.net/15d/. Accessed 14 Jan 2019.

[25] The 16D instrument. http://www.15d-instrument.net/16d-and-17d/16d/. Accessed 14 Jan 2019.

[26] The 15D instrument. Replacing missing data. http://www.15d-instrument.net/15d/replacing-missing-data/. Accessed 14 Jan 2019.

[27] World Health Organization (1992). The ICD-10 Classification of Mental and Behavioural Disorders: clinical descriptions and diagnostic guidelines.

[28] Helsinki and Uusimaa Hospital District. The HUS Clinical Trials Register. http://www.hus.fi. Accessed 14 Jan 2019.

[29] Bastiaansen D, Koot HM, Bongers IL, Varni JW, Verhulst FC (2004). Measuring quality of life in children referred for psychiatric problems: psychometric properties of the PedsQL 4.0 generic core scales. Qual Life Res..

[30] Jozefiak T, Larsson B, Wichstrom L, Wallander J, Mattejat F (2010). Quality of Life as reported by children and parents: a comparison between students and child psychiatric outpatients. Health Qual Life Outcomes..

[31] Mohler-Kuo M, Dey M (2012). A comparison of health-related quality of life between children with versus without special health care needs, and children requiring versus not requiring psychiatric services. Qual Life Res..

[32] Dey M, Mohler-Kuo M, Landolt MA (2012). Health-related quality of life among children with mental health problems: a population-based approach. Health Qual Life Outcomes..

[33] Coghill D, Hodgkins P (2016). Health-related quality of life of children with attention-deficit/hyperactivity disorder versus children with diabetes and healthy controls. Eur Child Adolesc Psychiatry..

[34] Bastiaansen D, Koot HM, Ferdinand RF (2005). Determinants of quality of life in children with psychiatric disorders. Qual Life Res..

[35] Lack CW, Storch EA, Keeley ML, Geffken GR, Ricketts ED, Murphy TK, Goodman WK (2009). Quality of life in children and adolescents with obsessive-compulsive disorder: base rates, parent–child agreement, and clinical correlates. Soc Psychiatr Epidemiol..

[36] Silberman MA, Snarey J (1993). Gender differences in moral development during early adolescence: the contribution of sex-related variations in maturation. Curr Psychol.

[37] Achenbach TM, Rescorla I. Manual of the ASEBA school-age forms & profiles: on integrated system of multi-informant assessment. Burlington, University of Vermont, Research Center for Children, Youth & Families: ASEBA; 2001.

[38] Helstelä L, Sourander A (2001). Self-reported competence and emotional and behavioral problems in a sample of Finnish adolescents. Nord J Psychiatry..

[39] Oshukova S, Kaltiala-Heino R, Miettunen J, Marttila R, Tani P, Aronen ET, Marttunen M, Kaivosoja M, Lindberg N (2016). The relationship between self-rated psychopathic traits and psychopathology in a sample of finnish community youth: exploration of gender differences. J Child Adolesc Behav..

[40] Depression. Current care guidelines. Working group set up by the Finnish Medical Society Duodecim and the Finnish Psychiatric Association. Helsinki: The Finnish Medical Society Duodecim. 2016. http://www.kaypahoito.fi. Accessed 14 Jan 2019.

[41] Conduct disorders. Current care guidelines. Working group appointed by the Finnish Medical Society Duodecim, The Finnish Society for Child and Adolescent Psychiatry, The Finnish Adolescent Psychiatric Association and The Section of Adolescent Psychiatry of the Finnish Psychiatric Association. Helsinki: The Finnish Medical Society Duodecim. 2018. http://www.kaypahoito.fi. Accessed 14 Jan 2019.

[42] Kaskeala L, Sillanmäki L, Sourander A (2015). Help-seeking behaviour among Finnish adolescent males. Nord J Psychiatry..

[43] Chaplin TM, Aldao A (2013). Gender differences in emotion expression in children: a meta-analytic review. Psychol Bull..

[44] Zahn Waxler C, Shirtcliff EA, Marceau K (2008). Disorders of childhood and adolescence: gender and psychopathology. Annu Rev Clin Psychol..

[45] Van der Graaff J, Carlo G, Crocetti E, Koot HM, Branje S (2018). Prosocial behavior in adolescence: gender differences in development and links with empathy. J Youth Adolescence..

[46] Balkin RS, Roland CB (2005). Identification of differences in gender for adolescents in crisis residence. J Ment Health..

[47] Weitkamp K, Daniels J, Rosenthal S, Romer G, Wiegand-Grefe S (2013). Health-related quality of life: cross-informant agreement of father, mother, and self-report for children and adolescents in outpatient psychotherapy treatment. Child Adolesc Ment Health..

[48] Karlsson L, Kiviruusu O, Miettunen J, Heila H, Holi M, Ruuttu T, Tuisku V, Pelkonen M, Marttunen M (2008). One-year course and predictors of outcome of adolescent depression: a case–control study in Finland. J Clin Psychiatry..

[49] Tuisku V, Kiviruusu O, Pelkonen M, Karlsson L, Strandholm T, Marttunen M (2014). Depressed adolescents as young adults—predictors of suicide attempt and non-suicidal self-injury during an 8-year follow-up. J Affect Disord..

